# Evaluation of cognitive functions in the elderly with and without central auditory processing disorder

**DOI:** 10.1590/2317-1782/20232022185en

**Published:** 2023-12-04

**Authors:** Caroline Nunes Rocha-Muniz, Tatiane Eisencraft Zalcman, Renata Alonso, Camila Maia Rabelo, Ivone Ferreira Neves-Lobo, Renata Filippini, Eliane Schochat

**Affiliations:** 1 Departamento de Fisioterapia, Fonoaudiologia e Terapia Ocupacional, Faculdade de Medicina, Universidade de São Paulo - USP - São Paulo (SP), Brasil.; 2 Faculdade de Ciências Médicas da Santa Casa de São Paulo - São Paulo (SP), Brasil.

**Keywords:** Aging, Auditory Perception, Cognition, Hearing, Elderly

## Abstract

**Purpose:**

Evaluate and compare the performance of cognitive functions between elderly with and without auditory processing disorders.

**Methods:**

Twenty-eight healthy elderly (14 Control group and 14 Auditory Processing Disorder group - APD group) participated in the study. All elderly were submitted to (central) auditory processing evaluation, P300 event-related potential and brief cognitive battery (BCB). In the comparison between subjects, the Mann-Whitney test was applied and in the intra-subjects comparison, the Wilcoxon signed rank test was used. To verify if there was an association between the performance of the PAC and the cognitive variables, Spearman's correlation was used.

**Results:**

There were no statistically significant differences between the Control and APD groups for the cognitive abilities assessed by BCB, as well as for the P300 cognitive potential variables.

**Conclusion:**

Elderly with auditory processing disorders do not seem to show greater cognitive difficulties compared to elderly of the same age group without auditory processing disorders, exception of the clock drawing test, whose task involves multiple cognitive functions, including visuospatial processing, executive function, semantic memory, and planning.

## INTRODUCTION

The effect of age on peripheral auditory function is well-known. Hearing losses related to the aging process are common among individuals older than 60 years old^(1)^.

However, there is some evidence that peripheral hearing losses in older adults are not entirely responsible for their comprehension difficulties. Anderson et al.^(2)^ demonstrated that even with similar hearing thresholds, younger and older adults present differences in the ability to understand speech in noise.

The aging process, along with hearing losses and structural and functional changes in the central nervous system, affects our listening, hearing, and auditory information processing. Thereby, several researchers have reported the influence of the aging process on different central auditory processing (CAP) skills^(3-7)^.

As many of these studies control the hearing sensitivity in different groups of individuals, such findings imply that the loss in peripheral sensitivity cannot explain all speech comprehension difficulties in older adults. Even older adults whose values of audiometric thresholds are within the normal standards can show alterations in the CAP linked to aging, which can be indicated through behavioral and/or electrophysiological measures^(5)^.

Some connections between CAP in older adults and subsequent dementia have been established. Even though many studies have concentrated on the relationship between peripheral hearing and subsequent dementia^(6)^, recent research has suggested that the CAP might indicate a stronger risk^(7)^.

Nevertheless, there is no consensus on the relationship between Central Auditory Processing Disorder (CAPD) and aging. There is a hypothesis more associated with a cognitive argument referring that changes in the perceptual processing of auditory information might be influenced by the decline in cognitive processes, that is, influenced by the top-down modulation. Changes in many forms of memory and attention have been observed. One of the universal findings is the lower speed of information processing (sensory and mental) with aging. Such a speech reduction in information processing, along with lower cognitive skills, affects listening comprehension^(8)^, especially in adverse or challenging environments, being linked to the work memory ability^(9)^.

Nonetheless, the relative contribution of cognitive factors to speech comprehension difficulty in older adults has been controversial. Arguments against a report of a more cognitive nature are based on the following findings:

1. The decline in cognitive functions linked to aging is highly correlated to concomitant alterations in peripheral sensitivity^(3)^.
2. Most attempts to associate changes in cognitive functions linked to aging, using basic measures of speech perception, have shown limited success^(10)^.

Briefly, the abovementioned studies support and confirm that older adults have speech comprehension difficulties compared with younger adults. Despite the effects of loss of high-frequency sensitivity for speech-in-silence perception to be understood, the lower speech comprehension among older adults in more complex and noisy listening situations seems to involve additional factors that cannot be predicted by the audiogram^(2)^. On the one hand, these findings are consistent with more cognitive models that describe a general reduction in mental processing speed. Older adults might evoke compensation strategies, such as the use of context or linguistic experience, to soften the effects of cognitive decline by communicating in more demanding environments^(8,9)^. There is also a possibility that during complex listening situations, the greater listening effort - resulting in age-related CAP declines aging - compromises the use of cognitive resources. According to Pichora-Fuller^(11:S59)^, “It is possible that at least some of the apparent differences linked to age in the cognitive performance during spoken language comprehension is secondary to the deficits in the temporal auditory processing”.

Considering such a controversy, the guiding question is: Would older adults with CAP alterations also present impaired cognitive functions?

To answer this question, in addition to assessing the cognitive functions of memory, attention, verbal fluency, and executive functions behaviorally, the use of electrophysiology, such as event-related auditory evoked potential assessment (ERP), can provide relevant information on the neurophysiological grounds of brain functioning^(12)^.

P300 is among the most investigated ERPs and can be seen as waves of positive polarity, with a maximum peak of approximately 300 ms, after the start of a relevant stimulus within the requested task. It can be raised by an oddball paradigm, which involves detecting and distinguishing a rare stimulus in a series of frequent stimuli. P300 originates from primary and secondary areas of the cortex, including the frontal and parietal regions. However, the exact trigger site is still unknown^(12)^.

Several studies have used the P300 as a non-invasive method that is sensitive to the monitoring of cognitive functions (including attention and memory), which might indicate a cognitive decline, as well as various neurodegenerative alterations^(13)^. The latency of the electrophysiological wave of the P300 can be considered a direct indicator of neural analysis of the stimulus and speed of processing by the individual, thus being considered a measure that represents the magnitude of the cognitive processing^(13)^. Changes in the latency and amplitude of the P300 might be a marker of cognitive declines associated with mild cognitive impairment (MCI) and Alzheimer’s disease^(13)^.

Considering the need to understand the issues associated with speech comprehension difficulty and aging, this study aimed to assess and compare the cognitive functions of older adults with and without CAP alteration. In addition, we investigated whether there is an association between performance in auditory processing tests and cognitive variables.

## METHODS

This is a cross-sectional, prospective, and observational study approved by the Ethics Committee, protocol number 0641/09. All participants were instructed regarding the research and signed the Informed Consent Form before the assessments.

### Case study

Twenty-eight healthy older adults participated in the study (60 - 79 years old). The volunteers belonged to a group of multidisciplinary outpatient geriatric care for older individuals and were invited according to their age, auditory history, and general health by professionals who work directly in the service, as well as by the researchers.

All participants presented hearing thresholds ≤ 25 dB at frequencies between 250 and 4000 Hz and ≤ 45 dB at frequencies between 6000 and 8000 Hz, no complaint of tinnitus, and performance within the normal range in both hearing aid and immittance.

None of the older adults presented alterations in the middle ear, asymmetrical sensorineural hearing loss, temporary or sudden hearing loss, history of vestibulocochlear disorders, or any neurological or psychiatric disorder. All of them had at least five years of formal education and none of them used medications that acted on the central nervous system.

Based on this initial sample, we scheduled and conducted the CAP assessments. According to the results, two groups were created: 1) the CAPD Group and 2) the Control Group.

The CAPD Group was composed of 14 older adults (mean ± standard deviation = 70.5 ± 4.69 years old) with complaints related to CAP difficulties, especially those linked to speech comprehension in noisy environments and competitive speech situations. Additionally, they presented an altered performance in the CAP assessment, which implies a performance below expected in at least two tests of the CAP assessment applied^(14)^. The CAP assessment was constituted of three tests - a monotonic test, a dichotic test, and a temporal processing test -, according to the recommendations of the American Speech-Language-Hearing Association (ASHA)^(14)^, as follows: a) Speech-in-white-noise test (SIN), at the signal-to-noise ratio + 20; b) Dichotic Digit Test (DD), at binaural integration^(15)^, and c) Pitch Pattern Sequence (PPS)^(16)^, which was applied binaurally. We analyzed the percentages of hits achieved by each individual in each test, both for the CAPD Group and the Control Group.

As normality values for the speech-in-noise test, we considered the signal-to-noise ratio +20dB and hits equal to or higher than 68% in the first tested ear, and equal to or higher than 72% in the second tested ear^(15)^. For the PPS, we adopted as normality a performance with hits equal to or higher than 75%^(16)^. In turn, for the DD test, the normality criterion corresponded to hits equal to or higher than 90% in both ears^(15)^. The normality values used herein follow the Assessment Manual of 1997, which has some differences from the normality values published in the Assessment Manual of 2011. Since the data collection was finished before the publication of the Assessment Manual of 2011, we decided to maintain the normality indicated by the Assessment Manual of 1997.

In turn, the Control Group was composed of 14 older adults without complaints or CAP assessment alterations (mean ± standard deviation = 67.93 ± 4.98 years old).

### Procedures

After the sample selection, all participants were subjected to a cognitive assessment using the Brief Cognitive Battery and an event-related electrophysiological test, the P300, which involves both physiological mechanisms related to CAP and cognitive functions. Both the tests were applied by speech therapists with a doctorate in the areas of neurology, audiology, and aging.

The Brief Cognitive Battery (BCB)^(17)^ is an instrument that assesses cognitive functions, including immediate memory, late memory, verbal fluency, and executive function. It has proven efficient at differentiating normal individuals with cognitive damage, including groups with distinct educational levels, which is the case with our population, including older adults.

The BCB requests that the individual identify and name ten objects in the following steps: 1) Incidental memory - recalling the objects immediately after the drawings are removed; 2) Immediate memory - recalling the objects a second time, after the subject had observed the objects for a further 30 seconds; 3) Learning - recalling the objects for a third time, after the subject had observed the objects for a further 30 seconds; 4) Late memory - after an interference period in which the categorical animal fluency tests (number of animals in one minute) and the Clock Drawing are applied, the individual is asked to recall the objects, and 5) Recognition - the individual is asked to recognize the objects originally presented, which are mixed among 10 other distracting drawings.

Each step is assigned a score that reflects the scoring of each participant. Such a score was used to analyze the BCB performance data.

The electrophysiological assessment was performed through the P300 event-related auditory potential.

The P300 was conducted using Navigator Pro equipment (by Biologic). The following parameters were used to obtain the P300: monaural acoustic stimuli of 500 Hz as frequent stimulus and 750 Hz as the rare stimulus (tone burst in a Blackman window, with a plateau of 30 cycles and rise/fall of 10 cycles); the intensity of both stimuli of 70 dB HL; analysis time of 800 ms; filter from 0.5 to 30 Hz; sensitivity of 100 µV. All stimuli were presented through an ER-3A insert earphone. We used 300 artifact-free stimuli, 80% of which were frequent and 20% rare. The rare and frequent stimuli were presented randomly (oddball paradigm) at a rate of 1.1 stimuli per second^(18)^.

The electrodes were placed on the vertex (Cz) and each side of the ear (A1 for the left ear and A2 for the right ear), with the “ground” electrode on the ear contralateral to the one being assessed^(19)^. The right and left ears were assessed separately.

The P300 was obtained by subtracting the trace corresponding to rare stimuli from the trace corresponding to frequent stimuli, which was identified as the wave with positive polarity with an approximate latency of 300 milliseconds (ms) post-stimulus. To analyze the P300 data, once it was present, we considered the wave latency (milliseconds) and amplitude (maximum wave point - minimum wave point/peak valley) of the wave^(18)^, for both the right ear and the left ear.

### Statistical analysis

The statistical method used was aimed at comparing the Control and CAPD groups for the performance of cognitive functions and verifying whether there was an association between the performances in the association tests and in the CAP tests and cognitive variables. For such a purpose, we conducted descriptive analyses of median, standard deviation, minimum, and maximum. Since the sample deviated from the normal distribution, we applied non-parametric tests. The Mann-Whitney test was used to compare the medians of the tests between the two studied groups. We also carried out an analysis between the ears (intra-subject) using the Wilcoxon signed rank test. The Spearman correlation was applied to verify whether there was an association between CAP performance and cognition. To interpret the magnitude of correlations, we adopted the following classification of correlation coefficients: correlation coefficients < 0.4 (correlation of weak magnitude), > 0.4 to < 0.5 (moderate magnitude), and > 0.5 (strong magnitude)^(20)^.

All statistical analyses were performed on the SPSS software (version 20.0) at a significance level of 5%.

## RESULTS

We analyzed the age, education (years of formal education), and sex variables both for the Control Group and the CAPD Group. As the data distribution for the ‘age’ and ‘education’ variables (Table 1) showed to be regular, we used the ANOVA parametric test to compare the values between the groups.

**Table 1:** Descriptive statistics of the values (in years) of the age and education variables for the Control and APD groups

	**Group**	**N**	**Median**	**SD**	**Min**	**Max**
**Age**	Control	14	67.50	4.98	60.00	75.00
	APD	14	70.00	4.69	62.00	79.00
					
**Education**	Control	14	11	3.52	4	15
	APD	14	8	3.81	2	15
					

**Caption:** N = Number of individuals, SD= Standard deviation, Min=Minimum, Max=Maximum, APD=Auditory Processing Disorder

The Control and CAPD groups showed no statistically significant difference either for the age variable [F (1.26)=1.98, p=0.17)] or the ‘education’ variable ( formal education years), [F(1.26)=0.45, p=0.51)].

The chi-square test was used to verify whether there was a difference in the sample proportion regarding the sex variable. The Control Group was composed of nine male individuals and eight female individuals [X^2^ (1, N = 14) = 1.14 p = 0.29]. In turn, the CAPD Group was composed of three male individuals and 11 female individuals [X^2^ (1. N = 14) = 4.57 p = 0.03].

As expected, the CAP behavioral tests showed that the Control Group achieved a better performance than the CAPD Group. The Mann-Whitney test indicated a statistically significant difference for the DD test both in the right ear (p=0.003*) and the left ear (p=0.008*), in addition to the PPS (p<0.001*) (Table 2).

**Table 2:** Descriptive statistics of the values (in percentage) obtained through the behavioral tests in the auditory processing assessment and p-value (Mann-Whitney test) for the comparison between the Control and APD groups

	**Group**	**N**	**Median**	**DP**	**Min**	**Max**	**p-value**
**SIN - RE**	Control	14	76	7.32	68.00	92.00	0.38
	APD	14	74	11.17	48.00	92.00	
						
**SIN - LE**	Control	14	80	7.43	72.00	96.00	0.21
	APD	14	76	7.94	64.00	92.00	
						
**DD - RE**	Control	14	100	1.63	95.00	100.00	0.003*
	APD	14	95	20.15	35.00	100.00	
						
**DD - LE**	Control	14	93.75	7.31	78.00	100.00	0.008*
	APD	14	85	22.00	37.50	97.50	
						
**PPS**	Control	14	80	7.70	75.00	100.00	<0.001*
	APD	14	25	21.70	15.00	100.00	
						

* Statistically significant difference

**Caption:** N = Number of individuals, DP= Standard deviation, Min=Minimum, Max=Maximum, APD=Auditory Processing Disorder, SIN=Speech-in-noise test, DD= Dichotic Digit Test, PPS= Pitch Pattern Sequence Test, RE = Right Ear, LE = Left Ear

When comparing the ears (intra-group analysis), the Wilcoxon signed rank test showed a statistically significant difference in the SIN test only for the Control Group (p=0.04*), which is a higher median of responses in the left ear than the median of responses in the right ear. In turn, the comparison between ears through the DD test showed a statistically significant difference both for the Control Group (p=0.008*) and the CAPD Group (p=0.01*).

As to the performance in the Brief Cognitive Battery, all individuals presented regular values, thus disregarding dementia. In addition, Table 3 shows that the performance of the group without processing alterations had slightly higher medians than the control group; however, no statistically significant difference was found. In turn, the Clock Drawing Test indicated a statistically significant difference between the groups, with the Control Group presenting a better performance compared with the CAPD Group (Table 3).

**Table 3:** Descriptive statistics of the values (number of scores) obtained through the Brief Cognitive Battery and p-value (Mann-Whitney test) for the comparison between the Control and APD groups

	**Group**	**N**	**Median**	**DP**	**Min**	**Max**	**p-value**
**Incidental Memory**	Control	14	5.5	1.34	4.00	9.00	0.454
	APD	14	5	1.05	5.00	8.00	
						
**Immediate Memory**	Control	14	8	1.12	6.00	10.00	0.104
	APD	14	9	1.28	6.00	10.00	
						
**Learning**	Control	14	8.5	0.99	7.00	10.00	0.125
	APD	14	9	0.61	8.00	10.00	
						
**Verbal Fluency**	Control	14	19	6.00	8.00	26.00	0.178
	APD	14	15	2.05	13.00	21.00	
						
**Clock Drawing**	Control	14	9.5	0.84	7.00	10.00	0.006*
	APD	14	9	1.20	5.00	9.00	
						
**Late Memory**	Control	14	9	1.45	6.00	10.00	0.482
	APD	14	9	0.73	8.00	10.00	
						
**Recognition**	Control	14	10	0.00	10.00	10.00	0.541
	APD	14	10	0.36	9.00	10.00	
						

* Statistically significant difference

**Caption:** N = Number of individuals, DP= Standard deviation, Min=Minimum, Max=Maximum, APD=Auditory Processing Disorder

The electrophysiological assessment (P300) found no statistically significant difference between the groups for none of the analyzed variables (Table 4).

**Table 4:** Descriptive statistics of the values de latency (ms) and amplitude (μV) were obtained through the cognitive potential P300 and p-value (Mann-Whitney test) for the comparison between the Control and APD groups

	**Group**	**N**	**Median**	**SD**	**Min**	**Max**	**p-value**
**P300 Latency RE**	Control	14	405.20	45.66	327.12	462.45	0.72
	APD	14	396.87	36.98	330.24	457.25	
						
**P300 Latency LE**	Control	14	364.59	39.28	321.91	448.92	0.89
	APD	14	378.13	39.55	299.01	430.18	
						
**P300 Amplitude RE**	Control	14	6.04	2.93	4.28	14.12	0.29
	APD	14	6.35	2.05	3.02	8.99	
						
**P300 Amplitude LE**	Control	14	6.67	3.95	3.34	17.80	0.27
	APD	14	5.43	2.89	2.35	12.26	
						

**Caption:** N = Number of individuals, SD= Standard deviation, Min=Minimum, Max=Maximum, APD=Auditory Processing Disorder, RE = Right ear, LE = Left ear

When investigating the association between the CAP tests and cognitive variables (Table 5), the Spearman correlation analysis showed a moderate correlation only between the Clock Drawing Test and the CAPD (r=0.43; p=0.024*). No significant correlations were found for the remaining relationships.

**Table 5:** Analysis of the Spearman correlation between the CAP variables and cognitive variables

		Incidental Memory	Immediate Memory	Learning	Verbal Fluency	Clock Drawing	Late Memory	Recognition
**SIN RE**	r	-0.004	-0.19	0.006	-0.03	0.17	0.09	-0.24
	p	0.98	0.34	0.977	0.89	0.39	0.66	0.22
	N	28	28	28	28	28	28	28
**SIN LE**	r	-0.19	-0.24	-0.07	-0.04	0.06	-0.28	-0.15
	p	0.33	0.22	0.724	0.82	0.76	0.15	0.45
	N	28	28	28	28	28	28	28
**DDT RE**	r	0.31	-0.35	-0.36	0.19	0.36	0.15	0.25
	p	0.11	0.06	0.06	0.34	0.06	0.44	0.2
	N	28	28	28	28	28	28	28
**DDT LE**	r	0.03	0.05	0.13	0.06	0.33	0.20	0.28
	p	0.86	0.80	0.52	0.75	0.09	0.31	0.15
	N	28	28	28	28	28	28	28
**PPS**	r	-0.1	0	0.15	0.37	**0.43**	0.01	0.27
	p	0.61	0.99	0.44	0.052	**0.024** *	0.62	0.17
	N	28	28	28	28	28	28	28

* Statistically significant difference

**Caption:** SIN=Speech-in-noise Test, DD= Dichotic Digit Test, PPS= Pitch Pattern Sequence Test, RE = Right Ear, LE = Left Ear; r = Coefficient of Correlation; p = p-value; N = Number of Individuals

## DISCUSSION

This study assessed and compared the cognitive functions of older adults with and without CAP alteration. Our results demonstrate that the group of older adults with CAPD (with performance below expected in CAP tests) presented no differences in most of the cognitive and executive functions assessed compared with healthy older adults (Control Group).

The comparison between the CAPD Group and the Control Group regarding the CAP behavioral tests showed a statistically significant difference between the DD and PPS Test Such a difference was not found in the speech-in-noise test.

Both the DD Test and the PPS Test involve an interhemispheric transfer through the corpus callosum. This structure is highly myelinated and has fibers for all sensory modalities, in addition to its role in modulating attention^(21)^ and verbal memory^(22)^. Aging can cause demyelination of the fibers, leading to a loss of integrity of the neural structures and influencing the speed of neural connections^(23)^. Thereby, considering our results, we might infer that some older adults can have alterations in the functioning of the corpus callosum, thus affecting the performance in tasks of binaural separation/integration and temporal and interhemispheric ordering, which are contained in the DD and PPS test.

When comparing the ears (intra-groups), we found a statistically significant advantage of the left ear (second tested ear) in the speech-in-noise test only for the Control Group. Such a result can be explained by the learning effect in older adults^(24)^. Furthermore, in general, low redundancy monaural tests indicate the learning effect for the second tested ear, and even though the pediatric/adult population does not show such an effect for the speech-in-noise test, it occurs in older adults^(16,24)^. In turn, the DD showed a statistically significant difference between the RE and the LE, both for the Control Group and the CAPD Group, with better performance for the RE.

Advantages of the RE over the LE are expected in dichotic tests that use verbal sounds, according to the model proposed by Kimura^(25)^. Based on this model, the RE has an advantage for verbal auditory stimuli because they are directly processed in the left hemisphere (the main hemisphere responsible for speech processing), through the contralateral pathways. When verbal stimuli are picked up in the left ear, they are first directed to the right hemisphere and then processed in the left hemisphere via the corpus callosum.

Another explanation for the asymmetry found in older adults (Control and CAPD) might be linked to a disadvantage of the left ear compared with the right ear. Such a disadvantage of the left ear due to aging has been reported in the literature^(26)^. Additionally, according to the theory that supports the DD test, stimulus processing in the left ear is required by the interhemispheric communication at the corpus callosum level^(16,27)^. Therefore, this potential disadvantage found in the left ear might result from a decreased functionality of the corpus callosum during aging, which is a fundamental structure for the interhemispheric required for the dichotic listening task^(27)^.

The absence of differences in performance in most of the cognitive tests of the brief battery between older adults with and without CAP alteration might suggest that despite the statistically significant differences found in the performance in CAP tests, the groups worked cognitively in the same way.

This proves to be an important result due to the controversy on how much the performance in CAP-related tasks is influenced by factors at higher cognitive levels, which might invalidate the CAP assessment results in older adults.

Nonetheless, an important result was observed for the Clock Drawing Test (CDT). Even though all research participants presented values within normality, thus disregarding dementia, the CAPD Group showed lower performance in the CDT compared with the Control Group, corresponding to a statistically significant difference.

The CDT has been widely used as a tool for neurological, psychiatric, and psychological assessment. Over the last decade, it has become increasingly more frequent as a fast, early screening tool for cognitive decline resulting from regular aging^(28)^.

Despite seeming a simple task, the CDT requires an accurate and successful engagement of multiple cognitive domains. By asking someone to draw a clock, we require them to understand the instructions, recover information linked to the concept of the clock using different types of memory processes, and translate such knowledge through visual-perceptual and visual-motor processes, in addition to assessing and monitoring the results of drawing through executive functions^(28)^. In addition, much of the information on the concept of a clock that must be remembered is abstract and conceptually complex. For example, the meaning of the spatial arrangement of the dial of a clock and the meaning of the hands with their different lengths. The multifactorial nature of the processes that underlie the act of drawing a clock is what makes this test highly sensitive to cognitive deficits^(29)^.

Thereby, we might infer that the sensory deficit (CAPD), in addition to the lower performance of the CDT by the CAPD Group compared with the Control Group, is one of the early stages in the aging process, hence preceding the onset of major cognitive deficits. Thus, the differences found between the groups only for the clock test might be the onset of cognitive difficulties.

We also highlight the association between the CDT and PPS tests. Originally, the PPS tests were developed to detect auditory consequences from brain lesions^(30)^ and have been categorized as tests that assess the temporal processing^(31)^. However, due to the acoustic features of the stimulus and the task required by the test (naming the tones and reproducing the sequence correctly), in addition to the influence of the acoustic properties of the stimulus, these tests are highly influenced by attention, work memory, and individual experiences^(32)^. Thus, the correlation between PPS and CDT found herein might be linked to such a cognitive demand of the PPS.

Murphy et al.^(33)^ also corroborate our findings by reporting an association between the Sustained Attention Test and the PPS.

In general, studies have demonstrated a correlation between hearing alterations and MCI, and/or Alzheimer’s disease^(34)^. The hypothesis for such a relationship suggests that the demand for the cognitive resources required to deal with a challenging auditory perception (as a result of hearing loss and/or hearing in adverse environmental conditions) might accelerate the neurocognitive decline during aging^(35)^.

In a sense, our results corroborate the study by Humes^(36)^, who investigated adults from 18 to 86 years old and found that middle-aged adults (until 55 years) had already experienced some decline, both in the sensory and the cognitive processing. The declines in these two domains are, for most of the measures taken by them, somewhere between young adults and older adults. This suggests that age-related declines in both domains are continuous and not abrupt in older adults. They also found a strong correlation between global sensory declines and cognitive declines. However, further research is needed, especially in the long term, to find out whether this relationship is causal and what the exact cause of this decline might be.

The electrophysiological assessment through the event-related potential (P300) showed no statistically significant differences between the Control and CAPD groups. Such an absence of difference between the groups might be linked to a similarity in the cognitive performance of the studied groups since the P300 is an electrophysiological measure related to cognitive processes.

Another explanation for the absence of a difference between the groups may be related to the peculiarities of this potential. Although the P300 reflects the speed and magnitude of neural processing of auditory stimuli, it is strongly influenced by many other features, in addition to cognitive and auditory factors, such as hormones, medication, and other aspects that are not related to the CAP^(37)^.

The sampling size is one of the limitations of this study, which might have influenced our results somehow, hence hindering a greater generalization for the population. Furthermore, other CAP skills that were not investigated herein, such as temporal resolution and binaural interaction, might also have influenced our results. Thereby, further research covering these issues must be conducted. We also highlight the need for long-term studies to monitor aging processes, both through cognitive tests and tests of CAP skills, to better establish the relationships between the bottom-up and top-down domains.

Despite the abovementioned limitations, by considering the set of evidence found herein and those reported in the literature, our results reinforce the theory that CAP alterations could precede cognitive alterations caused by the aging process. In addition, our study raises the importance of investigating hearing skills through CAP assessment in older adults to promote early intervention in aging processes, hence improving the quality of life for this population.

## CONCLUSION

This study aimed to assess and compare the performance of cognitive functions between older adults with and without CAP alteration. Herein, older adults with APD presented no alteration in most of the cognitive tests. Additionally, the performance of cognitive functions between older adults with and without CAP alterations showed to be similar, both through behavioral and electrophysiological assessment, except for the Clock Drawing Test, whose task involves multiple cognitive functions, including visual-spatial processing executive function, semantic memory, and planning. We could not verify the association between the performance in the CAP behavioral tests and the variables of cognitive functions, except for the PPS and the clock test.
