# Supplementary material for: Evaluation of cognitive functions in the elderly with and without central auditory processing disorder
Source: Codas. 2023 Dec 4;35(6):e20220185. doi: 10.1590/2317-1782/20232022185en (PMC10750824; doi:10.1590/2317-1782/20232022185en)
Supplement: Translation

# Translation: Avaliação das funções cognitivas em idosos com e sem alterações no processamento auditivo central

**DOI:** 10.1590/2317-1782/20232022185pt

**Authors:** Caroline Nunes Rocha-Muniz, Tatiane Eisencraft Zalcman, Renata Alonso, Camila Maia Rabelo, Ivone Ferreira Neves-Lobo, Renata Filippini, Eliane Schochat

**Affiliations:** 1 Departamento de Fisioterapia, Fonoaudiologia e Terapia Ocupacional, Faculdade de Medicina, Universidade de São Paulo – USP - São Paulo (SP), Brasil.; 2 Faculdade de Ciências Médicas da Santa Casa de São Paulo - São Paulo (SP), Brasil.

**Keywords:** Envelhecimento, Percepção Auditiva, Cognição, Audição, Idosos

## Abstract

**Objetivo:**

Avaliar e comparar as funções cognitivas entre idosos com e sem alteração do processamento auditivo central.

**Método:**

Vinte e oito idosos saudáveis (14 do grupo controle e 14 do grupo Transtorno do Processamento Auditivo Central - TPAC) participaram do estudo. Todos os idosos foram submetidos à bateria mínima de avaliação do processamento auditivo central (PAC), ao potencial auditivo cognitivo P300 e a bateria cognitiva breve (BCB). Na comparação entre sujeitos, foi aplicado o teste de Mann-Whitney e na comparação intra-sujeitos, utilizamos o teste de postos sinalizados de Wilcoxon. Para verificar se houve associação entre o desempenho do PAC e das variáveis cognitivas, foi utilizada a correlação de Spearman.

**Resultados:**

Não houve diferenças estatisticamente significantes entre os grupos ‘Controle’ e TPAC para a maioria das habilidades cognitivas avaliadas por meio da BCB, bem como para as variáveis do potencial cognitivo P300. Contudo, houve diferença estatisticamente significante entre o desempenho do grupo ‘Controle’ e TPAC para a prova do desenho do relógio. Além disso, foi possível observar correlação entre o desempenho do teste do relógio e no TPF.

**Conclusão:**

Idosos com TPAC não apresentaram alteração na maior parte dos testes cognitivos, com exceção da prova do desenho do relógio, cuja tarefa envolve múltiplas funções cognitivas, incluindo processamento visuoespacial, função executiva, memória semântica e planejamento.

## INTRODUÇÃO

O efeito da idade na função auditiva periférica é bem conhecido. As perdas auditivas relacionadas ao processo de envelhecimento são comuns entre indivíduos depois dos 60 anos^(1)^.

Contudo, há algumas evidências de que as perdas auditivas periféricas em idosos não são inteiramente responsáveis pelas dificuldades de compreensão. Anderson et al.^(2)^ demonstraram que, mesmo com limiares auditivos semelhantes, adultos mais jovens e mais velhos apresentaram diferença na habilidade para entender a fala no ruído.

Com o processo de envelhecimento, perdas auditivas, mudanças estruturais e funcionais no sistema nervoso central afetam como ouvimos, escutamos e processamos as informações auditivas. Assim, a influência do processo de envelhecimento em várias habilidades do processamento auditivo central (PAC) tem sido documentada por vários pesquisadores^(3-7)^.

Como muitos desses estudos controlam a sensibilidade auditiva em diferentes grupos de sujeitos, esses achados implicam que a perda da sensibilidade periférica não pode explicar todas as dificuldades de compreensão da fala entre os idosos. Mesmo idosos com limiares audiométricos com valores dentro dos padrões de normalidade podem demonstrar alterações no PAC relacionados com o avanço da idade, o que pode ser demonstrado por meio de medidas comportamentais e/ou eletrofisiológicas^(5)^.

Algumas conexões entre o PAC em idosos e demência subsequente tem sido estabelecidas. Embora muitos estudos tenham se concentrado na relação da audição periférica com uma demência subsequente^(6)^, pesquisas recentes sugerem que o PAC pode ser um indicador de risco mais forte^(7)^.

Apesar disso, não há um consenso entre um transtorno do processamento auditivo central (TPAC) e o envelhecimento. Há outra hipótese, mais associada há um argumento cognitivo, a qual refere que as mudanças no processamento perceptual das informações auditivas podem ser, na verdade, influenciadas pelo declínio de processos cognitivos, ou seja, influenciadas pela modulação *top-down*. São observadas mudanças em várias formas de memória e atenção. Um dos achados universais é a redução da velocidade do processamento da informação (sensorial e mental) com o avanço da idade. Essa redução na velocidade no processamento na informação, juntamente com a redução das habilidades cognitivas, afeta a compreensão auditiva^(8)^, especialmente em ambientes adversos ou desafiadores e tem sido relacionada com a capacidade da memória de trabalho^(9)^.

Entretanto, a contribuição relativa dos fatores cognitivos na dificuldade de compreensão de fala nos idosos tem sido controversa. Argumentos contra um relato mais cognitivo baseiam-se principalmente em descobertas que:

1. Declínios nas funções cognitivas relacionados ao envelhecimento estão altamente correlacionados com alterações concomitantes na sensibilidade periférica^(3)^.
2. A maioria das tentativas de associar as mudanças na função cognitiva, relacionadas ao envelhecimento, com medidas básicas de percepção da fala demonstraram sucesso limitado^(10)^.

Em resumo, as pesquisas levantadas acima suportam e confirmam que os idosos apresentam dificuldades de compreensão de fala, quando comparado a adultos mais jovens. Embora os efeitos da perda de sensibilidade de alta frequência na percepção da fala no silêncio sejam bem compreendidos, a redução na compreensão de fala entre idosos durante situações de escuta mais complexas e ruidosas parece envolver fatores adicionais não previsíveis pelo audiograma^(2)^. Por um lado, tais descobertas são consistentes com modelos mais cognitivos que descrevem uma redução geral na velocidade do processamento mental. Idosos podem invocar estratégias de compensação (por exemplo, uso de contexto ou experiência linguística) para diminuir os efeitos do declínio cognitivo ao se comunicar em ambientes mais exigentes^(8,9)^. Por outro lado, permanece a possibilidade de que, durante situações auditivas complexas, o aumento do esforço de escuta - resultante de declínios no PAC relacionados à idade - possa comprometer a utilização de recursos cognitivos. Como Pichora-Fuller^(11:S59)^ afirma, “é possível que pelo menos algumas das diferenças aparentes relacionadas à idade no desempenho cognitivo durante a compreensão da linguagem falada possam ser secundárias aos déficits do processamento temporal auditivo”.

Diante dessa controvérsia, a grande questão seria: Idosos com alteração de PAC apresentariam também comprometimento em funções cognitivas?

Para investigar essa pergunta, além de avaliar, por meio comportamental, as funções cognitivas referentes a memória, atenção, fluência verbal e funções executivas, a utilização da eletrofisiologia, como a avaliação de potenciais evocados auditivos relacionados a eventos (PEARE), pode trazer informações relevantes sobre os fundamentos neurofisiológicos do funcionamento do cérebro^(12)^.

O P300 é um dos PEARE mais investigados, sendo visualizado em forma de onda com polaridade positiva, com um pico máximo de aproximadamente 300 ms, após o início de um estímulo relevante dentro da tarefa solicitada. Pode ser eliciado por um paradigma *oddball*, o qual envolve a detecção e discriminação de um estímulo raro em uma série de estímulos frequente. O P300 é originado em áreas primárias e secundárias do córtex, incluindo as regiões frontal e parietal. Contudo, o exato sítio eliciador ainda é desconhecido^(12)^.

Vários estudos têm utilizado o P300 como um método não invasivo sensível para o monitoramento de funções cognitivas (incluindo atenção e memória) e pode indicar um declínio cognitivo, bem como em várias alterações neurodegenerativas^(13)^. A latência da onda eletrofisiológica do P300 pode ser considerada um indicador direto da análise neural do estímulo e da velocidade do processamento dessa análise pelo indivíduo, sendo considerado uma medida que representa a magnitude do processamento cognitivo^(13)^. Mudanças na latência e amplitude do P300 poderiam ser um marcador de declínios cognitivos associados com comprometimentos cognitivos leves (CCL) e doença de Alzheimer^(13)^.

Diante da necessidade de compreender as questões associadas entre as dificuldades de compreensão de fala e o envelhecimento, o objetivo desse estudo foi avaliar e comparar as funções cognitivas entre idosos com e sem alteração do PAC. Além disso, esse estudo verificou se há uma associação entre o desempenho nos testes de processamento auditivo e as variáveis cognitivas.

## MÉTODOS

O presente estudo transversal, prospectivo e observacional foi aprovado pelo comitê de ética, sob o protocolo de número 0641/09 Todos os participantes foram orientados sobre o estudo e assinaram o termo de consentimento livre e esclarecido antes das avaliações.

### Casuística

Vinte e oito idosos saudáveis participaram do estudo (60 – 79 anos de idade). Os voluntários pertenciam a um grupo de atendimento multidisciplinar ao idoso ambulatorial geriátrica e foram convidados por profissionais que trabalham diretamente no serviço, bem como, pelos próprios pesquisadores, de acordo com a idade, antecedentes auditivos e saúde geral de cada indivíduo.

Todos os participantes apresentavam limiares auditivos ≤ 25 dB nas frequências entre 250 e 4000 Hz e ≤ 45 dB nas frequências entre 6000 e 8000 Hz, sem queixa de tinnitus e desempenho dentro dos padrões de normalidade tanto na logoaudiometria quanto na imitanciometria.

Os idosos não possuíam alterações de orelha média, perdas auditivas neurossensoriais assimétricas, perdas auditivas temporárias e súbitas, antecedentes de afecções vestíbulo-cocleares ou qualquer alteração neurológica ou psiquiátrica e possuíam, no mínimo, cinco anos de educação formal. Além disso, nenhum dos idosos faziam uso de medicamentos com ação no sistema nervoso central.

A partir dessa amostra inicial, foram agendadas e realizadas as avaliações do PAC. De acordo com os resultados dessa avaliação, foram criados dois grupos: 1) Grupo com TPAC 2) Grupo Controle.

O grupo TPAC foi composto por 14 idosos (média ± desvio padrão = 70,5 ± 4,69 anos) que apresentavam queixas relacionadas a dificuldades do PAC, principalmente relacionados a compreensão de fala em ambientes ruidosos e em situações de fala competitiva. Além disso, apresentaram desempenho alterado na avaliação do PAC – ou seja, apresentaram desempenho abaixo do esperado em, ao menos, dois testes da avaliação do PAC aplicada^(14)^. A avaliação do PAC realizada foi composta de três testes, contemplando um teste monótico, um teste dicótico e um teste de processamento temporal, seguindo as recomendações da *American Speech-Language-Hearing Association (ASHA)* ^(14)^, a saber: a) teste de fala com ruído branco (FR), realizado na relação sinal/ruído + 20; b) teste dicótico de dígitos (DD), na modalidade de integração binaural^(15)^ c) teste padrão de frequência (TPF)^(16)^, o qual foi aplicado de forma binaural. Foram analisadas as porcentagens de acerto obtidas por cada indivíduo em cada teste, tanto no grupo TPAC quanto no grupo Controle.

Foram considerados valores de normalidade do teste de Fala com Ruído, na relação sinal/ruído +20dB, a obtenção da porcentagem de acertos igual ou superior a 68%, na primeira orelha testada, igual ou superior a 72% na segunda orelha testada^(15)^. Para o TPF, foi adotado como normalidade uma performance com porcentagem de acertos igual ou superior a 75%^(16)^. No teste DD, o critério de normalidade adotado, para esse estudo, foi a porcentagem de acertos igual ou superior a 90% em ambas as orelhas^(15)^. Os valores de normalidade utilizados nesse estudo estão de acordo com o Manual de Avaliação de 1997, que possui algumas diferenças em relação aos valores de normalidade publicados do Manual de Avaliação de 2011. Uma vez que a coleta dos dados foi finalizada anteriormente à publicação do Manual de Avaliação de 2011, optamos por manter a normalidade do Manual de Avaliação de 1997.

Já o grupo Controle foi composto por 14 idosos que não possuíam queixas ou alterações na avaliação do PAC (média ± desvio padrão = 67,93 ± 4,98 anos).

### Procedimentos

Após a seleção da amostra, todos os participantes foram submetidos a avaliação cognitiva utilizando a Bateria Cognitiva Breve e ao teste eletrofisiológico relacionado a eventos, P300, o qual envolve tanto mecanismos fisiológicos relacionados ao PAC quanto a funções cognitivas. Ambos os testes foram aplicados por fonoaudiólogos com doutorado nas áreas de neurologia, audiologia e envelhecimento.

A Bateria Cognitiva Breve (BCB)^(17)^ é um instrumento que avalia funções cognitivas, dentre elas, memória imediata, memória tardia, fluência verbal e função executiva. Essa bateria tem se mostrado eficiente ao diferenciar indivíduos normais daqueles com prejuízo cognitivo, inclusive em grupos com níveis educacionais heterogêneos, o que ocorre em nossa população, inclusive em idosos.

No BCB o indivíduo deve identificar e nomear 10 objetos em algumas etapas: 1) memória incidental – evocação dos objetos imediatamente após os desenhos serem retirados; 2) memória imediata – evocação dos objetos pela segunda vez, após o indivíduo observar os objetos por mais 30 segundos; 3) aprendizado - evocação dos objetos pela terceira vez, após o indivíduo observar os objetos por mais 30 segundos; 4) fase de evocação tardia - após um período de interferência em que são aplicados os testes de fluência categórica de animais (número de animais em um minuto) e o desenho do relógio, é solicitado a evocação dos objetos 5) fase de reconhecimento – é solicitado que o indivíduo reconheça os objetos originalmente apresentado, os quais estão misturados entre outros 10 desenhos distratores.

Para cada etapa realizada, é atribuído um escore que reflete a pontuação de acertos do indivíduo. Esse escore foi utilizado para a análise dos dados de desempenho no BCB.

A avaliação eletrofisiológica foi realizada por meio do potencial auditivo relacionado a eventos P300.

O P300 foi eliciado por meio equipamento Navigator Pro (marca Biologic). Os parâmetros utilizados para a aquisição do P300 foram os seguintes: estímulos acústicos monoaurais de 500 Hz para o estímulo frequente e 750 Hz para o raro (*tone burst* em uma janela Blackman, com plateau de 30 ciclos e rise/fall de 10 ciclos); intensidade de ambos os estímulos de 70 dBNA; tempo de análise de 800 ms; filtro de 0,5 a 30 Hz; sensibilidade de 100 µV. Os estímulos foram apresentados por meio de fone de inserção ER-3A. Foram utilizados 300 estímulos livres de artefatos, dentre os quais 80% eram frequentes e 20% eram raros. Os estímulos raros e frequentes foram apresentados de forma aleatória (paradigma oddball) na velocidade de 1,1 estímulos por segundo^(18)^.

Os eletrodos foram posicionados no vértex (Cz) e em cada um dos lados da orelha (A1 para a orelha esquerda e A2 para a orelha direita), estando o eletrodo “terra” na orelha contralateral à avaliada^(19)^. As orelhas, direita e esquerda, foram avaliadas separadamente.

O P300 foi obtido por meio da subtração do traçado correspondente aos estímulos raros em relação ao traçado correspondente aos estímulos frequentes, e foi identificado como a onda com polaridade positiva com latência aproximada de 300 milissegundos (ms) pós-estímulo. Para análise dos dados do P300, uma vez presente, foi considerada a análise de latência da onda (milissegundos) e amplitude (ponto máximo da onda – ponto mínimo da onda/ pico-vale) da onda (μV)^(18)^, tanto para a orelha direita quanto para a orelha esquerda.

### Análise estatística

Conforme os objetivos já especificados, o método estatístico utilizado visou à comparação dos grupos Controle e TPAC para o desempenho de funções cognitivas e verificar se houve associação entre o desempenho nos testes associação entre o desempenho nos testes de PAC e as variáveis cognitivas. Para isso, foram realizadas análises descritivas: mediana, desvio padrão, mínimo e máximo. Uma vez que a amostra apresentou desvios em relação a distribuição normal, foram aplicados testes não-paramétricos. Na comparação entre as medianas dos testes entre os dois grupos estudados, foi aplicado o teste de Mann-Whitney. Também foi realizada uma análise entre orelhas (intra-sujeito) utilizando o teste de postos sinalizados de Wilcoxon. Para verificar se havia associação entre o desempenho do PAC e das variáveis cognitivas, foi utilizada a correlação de Spearman. Para interpretar a magnitude das correlações adotamos a seguinte classificação dos coeficientes de correlação: coeficientes de correlação < 0,4 (correlação de fraca magnitude), > 0,4 a < 0,5 (moderada magnitude) e > 0,5 (forte magnitude)^(20)^.

As análises estatísticas foram realizadas por meio do software SPSS (versão 20.0) e o nível de significância estatística considerado foi de 5%.

## RESULTADOS

As variáveis idade, escolaridade (quantidade em anos de educação formal) e sexo, foram analisadas, tanto no grupo Controle quanto no grupo TPAC. Como a distribuição dos dados para as variáveis ‘idade’ e ‘escolaridade’ (Tabela 1) demonstraram uma distribuição normal, foi utilizado o teste paramétrico ANOVA para a comparação dos valores entre grupos.

**Tabela 1:** Estatística descritiva dos valores (em anos) das variáveis Idade e Escolaridade dos grupos Controle e TPA

	**Grupo**	**N**	**Mediana**	**DP**	**Min**	**Max**
**Idade**	Controle	14	67,50	4,98	60,00	75,00
	TPA	14	70,00	4,69	62,00	79,00
					
**Escolaridade**	Controle	14	11	3,52	4	15
	TPA	14	8	3,81	2	15
					

**Legenda:** N = Número de indivíduos, DP= Desvio Padrão, Min=Mínimo, Max=Máximo, TPA=Transtorno do Processamento Auditivo

O grupo Controle e TPAC não apresentaram diferença estatisticamente significante, tanto para a variável ‘idade’ [F (1,26)=1,98, p=0,17)], quanto para a ‘escolaridade’(anos de educação formal), [F(1,26)=0,45, p=0,51)].

Com relação a variável ‘sexo’, foi utilizado o teste qui-quadrado para verificar se houve diferença na proporção na amostra. O grupo controle foi composto por 9 indivíduos do sexo masculino e 8 do sexo feminino [X^2^ (1, N = 14) = 1,14 p = 0,29]. Já no grupo TPAC foi composto por 3 indivíduos do sexo masculino e 11 do sexo feminino [X^2^ (1, N = 14) = 4,57 p = 0,03].

Nos testes comportamentais do PAC, como esperado, o grupo Controle obteve melhor desempenho em comparação ao grupo TPAC. O teste de Mann-Whitney mostrou que houve diferença estatisticamente significante para o teste DD, tanto para orelha direita (p=0,003*) quanto para orelha esquerda (p=0,008*) e para o TPF (p<0,001*) (tabela 2).

**Tabela 2:** Estatística descritiva dos valores (em porcentagem) obtidos nos testes comportamentais da avaliação do processamento auditivo e p-valor (teste de Mann-Whitney) da comparação entre os grupos Controle e TPA

	**Grupo**	**N**	**Mediana**	**DP**	**Min**	**Max**	**p-valor**
**FR - OD**	Controle	14	76	7,32	68,00	92,00	0,38
	TPA	14	74	11,17	48,00	92,00	
						
**FR - OE**	Controle	14	80	7,43	72,00	96,00	0,21
	TPA	14	76	7,94	64,00	92,00	
						
**DD-OD**	Controle	14	100	1,63	95,00	100,00	0,003*
	TPA	14	95	20,15	35,00	100,00	
						
**DD-OE**	Controle	14	93,75	7,31	78,00	100,00	0,008*
	TPA	14	85	22,00	37,50	97,50	
						
**TPF**	Controle	14	80	7,70	75,00	100,00	<0,001*
	TPA	14	25	21,70	15,00	100,00	
						

* Diferença estatisticamente significante

**Legenda:** N = Número de indivíduos, DP= Desvio Padrão, Min=Mínimo, Max=Máximo, TPA=Transtorno do Processamento Auditivo, FR=Teste de Fala com ruído, DD= Teste Dicótico de Dígitos, TPF= Teste Padrão de Frequência, OD = Orelha Direita, OE = Orelha Esquerda

Na comparação entre orelhas (análise intra-grupo), o teste de postos sinalizados de Wilcoxon mostrou diferença estatisticamente significante no teste FR apenas para o grupo controle (p=0,04*), a mediana de respostas da orelha esquerda superior à mediana de respostas encontrada na orelha direita. Já para a comparação entre orelhas no teste DD, houve diferença estatisticamente significante tanto para o grupo controle (p=0,008*) quanto para o grupo TPAC (p=0,01*).

Em relação ao desempenho na Bateria Cognitiva Breve, todos os indivíduos apresentam valores dentro da normalidade, descartando o quadro de demência. Além disso, na Tabela 3, podemos verificar que o desempenho do grupo com alteração de processamento apresentou medianas discretamente maiores em comparação ao grupo ‘controle’, contudo, sem diferença estatisticamente significante. Entretanto, na prova do ‘Relógio’ observou-se diferença estatisticamente significante entre os grupos, sendo que o grupo ‘Controle’ apresentou melhor desempenho em relação ao grupo ‘TPAC’ (Tabela 3).

**Tabela 3:** Estatística descritiva dos valores (número do escores) obtidos na Bateria Cognitiva Breve e p-valor (teste de Mann-Whitney) da comparação entre os grupos Controle e TPA

	**Grupo**	**N**	**Mediana**	**DP**	**Min**	**Max**	**p-valor**
**Memória Incidental**	Controle	14	5,5	1,34	4,00	9,00	0,454
	TPA	14	5	1,05	5,00	8,00	
						
**Memória Imediata**	Controle	14	8	1,12	6,00	10,00	0,104
	TPA	14	9	1,28	6,00	10,00	
						
**Aprendizado**	Controle	14	8,5	0,99	7,00	10,00	0,125
	TPA	14	9	0,61	8,00	10,00	
						
**Fluência Verbal**	Controle	14	19	6,00	8,00	26,00	0,178
	TPA	14	15	2,05	13,00	21,00	
						
**Desenho do Relógio**	Controle	14	9,5	0,84	7,00	10,00	0,006*
	TPA	14	9	1,20	5,00	9,00	
						
**Memória Tardia**	Controle	14	9	1,45	6,00	10,00	0,482
	TPA	14	9	0,73	8,00	10,00	
						
**Reconhecimento**	Controle	14	10	0,00	10,00	10,00	0,541
	TPA	14	10	0,36	9,00	10,00	
						

* Diferença estatisticamente significante

**Legenda:** N = Número de indivíduos, DP= Desvio Padrão, Min=Mínimo, Max=Máximo, TPA=Transtorno do Processamento Auditivo

Já para a avaliação eletrofisiológica (P300) não houve diferença estatisticamente significante entre os grupos para nenhuma das variáveis analisadas (Tabela 4).

**Tabela 4:** Estatística descritiva dos valores de latência (ms) e amplitude (μV) obtidos no potencial cognitivo P300 e p-valor (teste de Mann-Whitney) da comparação entre os grupos Controle e TPA

	**Grupo**	**N**	**Mediana**	**DP**	**Min**	**Max**	**p-valor**
**P300 Latência OD**	Controle	14	405,20	45,66	327,12	462,45	0,72
	TPA	14	396,87	36,98	330,24	457,25	
						
**P300 Latência OE**	Controle	14	364,59	39,28	321,91	448,92	0,89
	TPA	14	378,13	39,55	299,01	430,18	
						
**P300 Amplitude OD**	Controle	14	6,04	2,93	4,28	14,12	0,29
	TPA	14	6,35	2,05	3,02	8,99	
						
**P300 Amplitude OE**	Controle	14	6,67	3,95	3,34	17,80	0,27
	TPA	14	5,43	2,89	2,35	12,26	
						

**Legenda:** N = Número de indivíduos, DP= Desvio Padrão, Min=Mínimo, Max=Máximo, TPA=Transtorno do Processamento Auditivo, OD = Orelha Direita, OE = Orelha Esquerda

Na investigação sobre a associação entre os testes de PAC e as variáveis cognitivas (tabela 5), a análise da correlação de Spearman mostrou correlação de moderada magnitude somente entre o teste do relógio e o TPF (r=0,43; p=0,024*). Nas demais relações, não foram encontradas correlações significativas.

**Tabela 5:** Análise da correlação de *Spearman* entre as variáveis do PAC e das variáveis cognitivas

		Memória Incidental	Memória Imediata	Aprendizado	Fluência Verbal	Desenho do Relógio	Memória Tardia	Reconhecimento
**FR OD**	r	-0,004	-0,19	0,006	-0,03	0,17	0,09	-0,24
	p	0,98	0,34	0,977	0,89	0,39	0,66	0,22
	N	28	28	28	28	28	28	28
**FR OE**	r	-0,19	-0,24	-0,07	-0,04	0,06	-0,28	-0,15
	p	0,33	0,22	0,724	0,82	0,76	0,15	0,45
	N	28	28	28	28	28	28	28
**TDD OD**	r	0,31	-0,35	-0,36	0,19	0,36	0,15	0,25
	p	0,11	0,06	0,06	0,34	0,06	0,44	0,2
	N	28	28	28	28	28	28	28
**TDD OE**	r	0,03	0,05	0,13	0,06	0,33	0,20	0,28
	p	0,86	0,80	0,52	0,75	0,09	0,31	0,15
	N	28	28	28	28	28	28	28
**TPF**	r	-0,1	0	0,15	0,37	**0,43**	0,01	0,27
	p	0,61	0,99	0,44	0,052	**0,024** *	0,62	0,17
	N	28	28	28	28	28	28	28

* Diferença estatisticamente significante

**Legenda:** FR=Teste de Fala com ruído, DD= Teste Dicótico de Dígitos, TPF= Teste Padrão de Frequência, OD = Orelha Direita, OE = Orelha Esquerda; r = Coeficiente de Correlação; p = p-valor; N = Número de Indivíduos

## DISCUSSÃO

O presente estudo avaliou e comparou as funções cognitivas entre idosos com e sem alteração do PAC. Nossos resultados demonstraram que o grupo de idosos com TPAC (ou seja, que desempenho abaixo do esperado em testes do PAC) não demonstraram diferenças na maioria das funções cognitivas e executivas avaliadas quando comparados a idosos saudáveis (Grupo Controle).

Na comparação entre o grupo TPAC e o grupo Controle, em relação aos testes comportamentais do PAC, observou-se diferença estatisticamente significante entre os testes DD e TPF. A mesma diferença não foi observada para o teste FR.

Tanto o DD quanto o TPF envolvem a transferência inter-hemisféricas por meio do corpo caloso. Essa estrutura é altamente mielinizada e possui fibras de todas as modalidades sensoriais, além de seu envolvimento na modulação da atenção^(21)^ e na memória verbal^(22)^. O envelhecimento pode causar a desmielinização das fibras levando a perda da integridade das estruturas neurais e influenciando na velocidade das conexões neurais^(23)^. Desta forma, considerando os resultados apresentados no presente estudo, nós podemos inferir que alguns idosos podem apresentar alteração em relação ao funcionamento do corpo caloso, afetando o desempenho em tarefas de separação/integração binaural e ordenação temporal e transferência inter-hemisférica as quais estão envolvidas no DD e TPF.

Na comparação entre orelhas (intra-grupos) foi observada vantagem estatisticamente significante da orelha esquerda (segunda orelha testada) para o teste FR apenas no grupo Controle. Este resultado pode ser explicado pelo efeito de aprendizado em idosos^(24)^. Além disso, de forma geral, os testes monoaurais de baixa redundância apresentam o efeito aprendizagem para a segunda orelha testada – e por mais que na população pediátrica/adulta esse efeito não seja observado para o teste fala com ruído, nos idosos esse efeito pode ser observado^(16,24)^. Já para o teste DD, foi observado diferença estatisticamente significante entre o desempenho da OD e OE, tanto para o grupo Controle quanto para o grupo TPAC, sendo observado melhor desempenho para OD.

Vantagens na OD sobre a OE são esperadas em testes dicóticos que utilizam sons verbais, de acordo com o modelo proposto por Kimura^(25)^. Segundo esse modelo, a vantagem da OD para estímulos auditivos verbais ocorre porque são diretamente processados no hemisfério esquerdo (principal hemisfério responsável pelo processamento da fala), por meio da atuação das vias contralaterais. Quando os estímulos verbais são captados na orelha esquerda, dirigem-se primeiramente ao hemisfério direito, para posteriormente, via corpo caloso, serem processados no hemisfério esquerdo.

Outra possibilidade que poderia explicar a assimetria encontrada nos idosos (Controle e TPAC), poderia estar relacionada à uma desvantagem da orelha esquerda em relação à orelha direita. A desvantagem da orelha esquerda com o envelhecimento tem sido reportada^(26)^. Além disso, de acordo com a teoria que subjaz o teste DD, o processamento do estímulo da orelha esquerda faz-se necessário a comunicação inter-hemisférica em nível de corpo-caloso^(16,27)^. Desta forma, essa possível desvantagem observada na orelha esquerda pode ser resultado de uma funcionalidade diminuída do corpo caloso no envelhecimento, estrutura fundamental para a transferência inter-hemisférica necessária para a tarefa de escuta dicótica^(27)^.

A ausência de diferenças no desempenho da maioria dos testes cognitivos da bateria breve entre os idosos com e sem alteração do PAC poderia sugerir que apesar das diferenças estatisticamente significantes encontradas no desempenho dos testes de PAC, cognitivamente, os grupos funcionavam da mesma forma.

Esse resultado mostra-se importante uma vez que ainda há controvérsias sobre o quanto o desempenho em tarefas relacionadas ao PAC é influenciado por fatores em níveis cognitivos mais elevados, os quais poderiam invalidar os resultados da avaliação do PAC em idosos.

Contudo, um resultado importante foi observado para o Teste do Relógio (TR). Apesar de todos os indivíduos da pesquisa apresentarem valores dentro da normalidade, descartando o quadro de demência, foi possível verificar que o grupo TPAC apresentou menor desempenho no TR em comparação ao grupo Controle, sendo essa diferença estatisticamente significante.

O TR tem sido largamente usado como ferramenta de avaliação neurológica, psiquiátrica e psicológica. Na última década tornou-se mais frequente o seu uso como ferramenta no rastreio rápido e precoce do declínio cognitivo decorrente do envelhecimento normal^(28)^.

Apesar do TR parecer uma tarefa simples, exige o envolvimento preciso e bem-sucedido de múltiplos domínios cognitivos. Ao pedirmos a uma pessoa que desenhe um relógio, estamos exigindo que ela compreenda as instruções, consiga recuperar informação relacionada com o conceito de relógio utilizando diferentes tipos de processos de memória, que traduza este conhecimento através de processos visuopercetivos e visuomotores, e ainda que consiga avaliar e monitorizar através das funções executivas o resultado que vai obtendo ao desenhar^(28)^. Além disto, muita da informação sobre o conceito de relógio que deve ser recordada é abstrata e conceitualmente complexa, por exemplo, o significado da disposição espacial do mostrador de um relógio e o significado dos ponteiros com seus diferentes comprimentos. A natureza multifatorial dos processos que subjazem o ato de desenhar um relógio é o que torna esse teste altamente sensível a déficits cognitivos^(29)^.

Desta forma, nós poderíamos especular que o déficit sensorial (TPAC), além de um menor desempenho no TR apresentado pelo grupo TPAC, em comparação ao grupo Controle, poderia ser um dos primeiros estágios no processo de envelhecimento e consequentemente, anterior ao desenvolvimento dos déficits cognitivos maiores. Assim, as diferenças encontradas entre os grupos somente para a prova do relógio poderia ser o início das dificuldades cognitivas que podem começar a surgir.

Além desse resultado, também ressaltamos a associação apresentada entre o teste TR e o TPF.

Originalmente, oTPF foi desenvolvido para verificar consequências auditivas em lesões cerebrais^(30)^ e tem sido categorizado como testes que avaliam o processamento temporal^(31)^. Contudo, devido às características acústicas do estímulo e a tarefa requerida pelo teste (nomeação dos tons e reprodução correta da sequência apresentada), além da influência das propriedades acústicas do estímulo, esses testes são muito influenciados pela atenção, memória de trabalho e experiências vivenciadas pelo indivíduo^(32)^. Desta forma, a correlação entre o TPF e o TR, encontrada no presente estudo, pode estar relacionada a essa demanda ‘cognitiva’ do TPF.

Outro estudo que corrobora nossos achados é Murphy et al.^(33)^, que demonstrou associação entre o teste de Atenção Sustentada e o TPF.

De forma geral, estudos tem demonstrado correlação entre alterações auditivas e CCL e/ou doença de Alzheimer^(34)^. A hipótese para essa relação sugere que a demanda dos recursos cognitivos necessários para lidar com uma percepção auditiva desafiadora (consequência da perda auditiva e/ou audição em condições ambientalmente adversas) poderia acelerar o declínio neurocognitivo durante o envelhecimento^(35)^.

Nossos resultados, de certa forma, corroboram o estudo de Humes^(36)^ que investigaram adultos de 18 a 86 anos de idade e que indicou que os adultos de meia idade (até os 55 anos) já experimentam declínios, tanto no processamento sensorial quanto no cognitivo. Os declínios nesses dois domínios situam-se, para a maioria das medidas por eles realizadas, em algum lugar entre os adultos jovens e os mais velhos. Isso sugere que os declínios relacionados à idade, em ambos os domínios, são contínuos e não abruptos nos idosos. Encontraram também uma forte correlação entre declínios sensoriais globais e declínios cognitivos, mas que mais pesquisas precisam ser realizadas, especialmente as longitudinais, para saber se essa relação é causal e qual seria a causa exata desse declínio.

Na avaliação eletrofisiológica por meio do potencial relacionado a eventos (P300), não houve diferenças estatisticamente significantes entre os grupos Controle e TPAC. Uma possível explicação para a ausência dessa diferença entre os grupos pode estar relacionada a semelhança no desempenho cognitivo entre os grupos estudados, uma vez que o P300 é uma medida eletrofisiológica relacionada a processos cognitivos.

Outra explicação para a ausência dessa diferença entre os grupos pode estar relacionada as peculiaridades desse potencial. Embora o P300 reflita a velocidade e a magnitude do processamento neural para o estímulo auditivo, mostra-se fortemente influenciado por muitos outros fatores, além dos fatores cognitivos e auditivos, como hormonais, medicamentosos entre outros fatores que não estão diretamente relacionados ao PAC^(37)^.

Uma limitação do estudo refere-se ao tamanho amostral reduzido, que pode ter impactado de alguma forma os resultados apresentados e desta forma, impede a generalização maior desses resultados para a população. Além disso, outras habilidades do PAC que não foram investigadas pelo presente estudo, como resolução temporal e interação binaural, também podem ter impactado os resultados apresentados no estudo. Por isso, novas pesquisas que contemplem essas questões devem ser realizadas. Também reforçamos a necessidade de pesquisas longitudinais, buscando acompanhar os processos de envelhecimento, tanto por meio de testes cognitivos quanto testes das habilidades de PAC e assim, tentar estabelecer melhor as relações entre os domínios bottom-up e top-down.

Apesar das limitações supracitadas, ao considerarmos, em conjunto, as evidências encontradas por esse estudo e as encontradas na literatura, nossos resultados reforçam a teoria de que a alteração do PAC poderia anteceder as alterações cognitivas causadas pelo processo de envelhecimento. Além disso, esse estudo levanta a importância da investigação das habilidades auditivas por meio da avaliação do PAC em idosos, a fim de promover intervenção precoce nos processos de envelhecimento e assim, melhorar a qualidade de vida dessa população.

## CONCLUSÃO

O objetivo do presente estudo foi avaliar e comparar o desempenho das funções cognitivas entre idosos com e sem alteração do PAC. Assim, no presente estudo, idosos com TPAC não apresentaram alteração na maior parte dos testes cognitivos. Além disso, o desempenho das funções cognitivas entre idosos com e sem alteração do PAC mostra-se semelhante, tanto por meio de avaliação comportamental quanto eletrofisiológica, com exceção da prova do desenho do relógio, cuja tarefa envolve múltiplas funções cognitivas, incluindo processamento visuoespacial, função executiva, memória semântica e planejamento. Não foi possível verificar associação entre o desempenho dos testes comportamentais do PAC e as variáveis de funções cognitivas, com exceção do TPF e a prova do relógio.
